# Tropical Hygiene

**Published:** 1898-05-07

**Authors:** 


### Tropical Hygiene.

The President of the United States is credited with having come to the very wise resolution to send none but "salted" troops to Cuba, that is, to select specially for military service in that country those who have already had yellow fever, and have thus become immune to its attacks.

Nothing can be more disastrous, from a military point of view, or more calamitous in regard to loss of life, than that a well-planned expedition should be thrown on its back and rendered useless by an outbreak of yellow fever, a disease which has probably been responsible for far more deaths among the Spanish troops employed in that unhappy island than have been caused by all the conflicts with all the insurgents put together. Still, with a practised and well organised sanitary service, we have no doubt that even yellow fever could be controlled, and, in fact, the belief is steadily growing that many of the dangers of the tropics could be avoided by hygienic precautions. This, however, is a hypothesis which has its limitation.

A paper which was read last week by Dr. Sambon before the Royal Geographical Society, on "Acclimatisation of the White Man in Tropical Lands," showed what wonderful changes in the healthiness of all tropical countries have been wrought within recent times by sanitation. In past times, he said, heat had been looked upon as the main factor in what was called climate, and heat had been supposed to induce deterioration of the race, and to cause such diseases as anæmia, liver abscess, and sunstroke. He pointed out, however, that many of the tropical diseases which were commonly looked upon as the causes of that deterioration of the white races which took place in hot countries, were really microbic in origin, and were thus capable of being to a large extent held in check by sanitary measures. The deterioration of the race was, he thought, far more alarming in the overcrowded cities of the old world than in tropical colonies, where, under proper management, even European children did very well. Even in the most unhealthy of these colonies the infant mortality was lower than in some districts in Europe.

Dr. Sambon also said that the old belief that white men could not labour in the tropics was disproved by facts, and he was sure that, although attempts at tropical colonisation had often been unsuccessful and had always cost immense sacrifices of life, this was due to their having been made in ignorance of the conditions essential to success, for acclimatisation was a mere matter of hygiene.

Probably Dr. Sambon is right, and, perhaps, in some future times we may find it possible to bring hygiene to such a pitch of perfection as to make residence in the tropics safe. It will not do, however, to make light of the magnitude of the task, for it is an enormous one. In cold countries sanitary measures have, so far, chiefly had for their aim the protection of man from disease propagated by man. In regard to the great mass of the diseases of temperate climes, which are recognised as infective, the infective agents are not capable of prolonged activity outside the body, so that, if our sanitation can protect us from the infections derived from other men, we are fairly safe. Quite otherwise is it, however, in the tropics, where in the presence of heat and moisture and an infinite supply of organic matter, lowly forms of life exist in endless variety, ready at any moment to swarm in man's living tissues, and thus to produce disease. Maladies so caused cannot be kept at bay by those ordinary precautions against filth, which in colder latitudes form the very groundwork of our hygienic faith.

Back to nature has here been our cry. Pure air and virgin soil, and water untainted by sewage products, have been looked upon as panaceas for all the ills of civilisation. But in the tropics the virgin soil and the pure air of an untouched country, where man has never placed his foot, may be teeming with organisms, such as those of malaria and many other parasitic maladies, quite as capable of producing disease as the more definitely human infections to which we in higher latitudes are accustomed, but quite incapable of being kept under control by those precautions which are so effective in the management of epidemics in more temperate climes. Hygiene in the tropics has quite a different meaning to that which the word conveys at home, and it would be reckless to suggest that by carrying out our own well-accepted principles people in the tropics can at once make themselves safe. As things exist at the present time, acclimatisation means, we think, something much more than mere hygiene, and a man's most potent protection against many tropical diseases is that immunisation which he gains from having had them, maybe in slight form, and thus of having become "well salted."

